# Tickborne Lymphadenopathy Complicated by Acute Myopericarditis, Spain

**DOI:** 10.3201/eid2112.150672

**Published:** 2015-12

**Authors:** José Tiago Silva, Francisco López-Medrano, Mario Fernández-Ruiz, Elena Resino Foz, Aránzazu Portillo, José A. Oteo, José Maria Aguado

**Affiliations:** Instituto de Investigación Hospital “12 de Octubre” (i+12), Madrid, Spain (J.T. Silva, F. López-Medrano, M. Fernández-Ruiz, E.R. Foz, J.M. Aguado);; Centro de Investigación Biomédica de La Rioja, Logroño, Spain (A. Portillo, J.A. Oteo)

**Keywords:** DEBONEL/TIBOLA/SENLAT, myopericarditis, tickborne rickettsiosis, vector-borne infections, ticks, Spain, tickborne lymphadenopathy, rickettsia, bacteria

**To the Editor:** *Dermacentor*-borne necrosis erythema lymphadenopathy/tickborne lymphadenopathy (DEBONEL/TIBOLA) is an apparently benign, self-limiting rickettsial disease transmitted by *Dermacentor* ticks (*1*,*2*). *Rickettsia slovaca* was the first etiologic agent isolated, but other species, such as *R. raoultii* and *Candidatus *R. rioja, also might be involved (*3*–*6*). If the scalp is affected, a larger number of agents (including *Francisella tularensis*, *Bartonella henselae*, *R. massiliae*, *R. sibirica mongolitimonae*, and *Borrelia burgdorferi*) should be considered within the differential diagnosis of a similar syndrome recently named scalp eschar associated with neck lymphadenopathy after a tick bite (SENLAT) (*7*). Nevertheless, in Spain, only *R. slovaca*, *Candidatus R. rioja*, and *F. tularensis* are known to cause DEBONEL/TIBOLA/SENLAT (*4*,*6*). This entity is considered an emerging rickettsiosis in Europe; cases have been reported from Italy, France, Hungary, Germany, and Portugal (*8*).

We recently saw a patient in whom acute myopericarditis developed after he was bitten by a large tick on the scalp and showed clinical signs of DEBONEL/TIBOLA/SENLAT, most likely attributable to *R. slovaca* or *Candidatus *R. rioja infection. The patient, a previously healthy 28-year-old man, went on a day-long hiking trip to the northern mountains of Madrid (central Spain; mean altitude 1,300 m) on November 2, 2014. Three days later, he noticed a mild ache on the occipital area of his scalp and found an attached tick that he removed with his fingers. A week later, he sought care from an infectious disease specialist because of itchy discomfort at the area of the tick bite. 

Examination revealed an erythematous and elevated punctiform lesion with mild fluctuation in the occipital region accompanied by tender, small lymph node enlargement of both occipital lymphatic chains (Figure). No widespread rash was present. DEBONEL/TIBOLA/SENLAT was diagnosed, and doxycycline (100 mg every 12 hours) was initiated. IgG titer against spotted fever group *Rickettsia* (SFGR) was 1:128. Four days later, the patient sought care at an emergency department, reporting retrosternal chest pain. Electrocardiogram revealed a diffuse ST-segment elevation with PR-segment depression; serum creatine phosphokinase and troponin T levels were 327 IU/L (reference range 10–190 IU/L) and 420 ng/mL (reference <14 ng/mL), respectively. Myopericarditis was diagnosed. A transthoracic echocardiogram ruled out pericardial effusion, valve vegetations, and left ventricular dysfunction; cardiovascular magnetic resonance imaging performed 4 days later showed myocardial inflammation. Blood cultures were sterile, pneumococcal urinary antigen test result was negative, and IgM against coxsackievirus and *Mycoplasma pneumoniae* were not detected. Nonsteroidal antiinflammatory drugs were prescribed. The patient improved clinically, and electrocardiogram findings resolved. The patient received doxycycline for 4 weeks.

**Figure:**
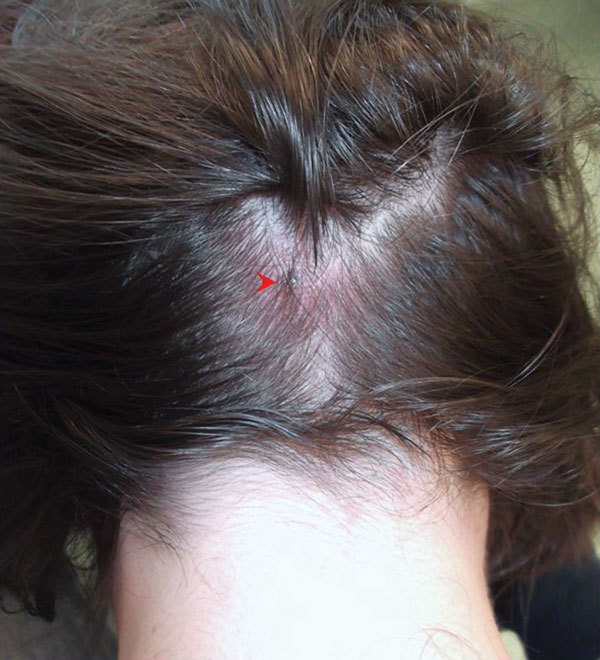
*Dermacentor*-borne necrosis erythema lymphadenopathy/tickborne lymphadenopathy/scalp eschar associated with neck lymphadenopathy after a tick bite. Shown is an erythematous, punctiform lesion in the scalp (arrow), accompanied by enlarged occipital lymph nodes

On a convalescent-phase serum specimen collected after 8 weeks, indirect immunofluorescence assays (IFA) for IgG against SFGR were performed in Spain’s national reference center for rickettsioses (Hospital San Pedro–Centro de Investigación Biomédica de La Rioja [CIBIR], Logroño, Spain). Commercial (Focus Diagnostics, Cypress, CA, USA) and in-house *R. conorii*, *R. slovaca*, and *R. raoultii* antibody testing showed an IgG titer of 1:512 against the 3 species. A subsequent cross-adsorption assay using *R. slovaca*, *R. raoultii*, and *R. conorii* antigens prepared on the basis of strains from the collection at Hospital San Pedro-CIBIR showed a decrease in IgG titers against *R. conorii* and *R. raoultii* to 1:64 and 1:256, respectively, whereas titer against *R. slovaca* remained at 512. IFA against *Bartonella* spp. and *C. burnetii* (Focus Diagnostics), chemiluminescence immunoassay for *B. burgdorferi* (Liason, DiaSorin, Spain), and in-house microagglutination assay for *F. tularensis* were not reactive. The patient recovered, with only a residual scarring alopecia on the occipital region of the scalp and without cardiac dysfunction after 9-month follow-up.

Myopericarditis is a rare complication of rickettsiosis, usually associated with *R. rickettsii* and *R. conorii* (*9*). Although tetracycline-induced cardiac adverse reactions have been described (*10*) and the patient reported here had signs of myopericarditis shortly after the initiation of doxycycline, he completed a 4-week treatment without recurrence. Therefore, the clinical picture seems unlikely to be attributable to doxycycline-induced toxicity. Because the patient was bitten in November (when only *Dermacentor* spp. ticks are active in central Spain), we have further epidemiologic evidence for attributing the infection to SFGR causing DEBONEL/TIBOLA/SENLAT. After serum adsorption, IFA titer against *R. slovaca* was 3-fold higher than that against *R. conorii*. *R. slovaca* and *Candidatus *R. rioja are the species most commonly found in *D. marginatus* ticks and in cases of DEBONEL/TIBOLA/SENLAT in Spain (*8*).

In view of the seroconversion to *Rickettsia* spp. with negative test results for other possible causative agents and the clinical response to doxycycline, rickettsiosis caused by *R. slovaca* or *Candidatus *R. rioja remains the most probable diagnosis. Because DEBONEL/TIBOLA/SENLAT is an emerging disease, physicians should consider that this entity may be associated with systemic complications similar to those of other tickborne rickettsioses.
